# Occupational recovery of Dutch workers with low back pain

**DOI:** 10.1093/occmed/kqac067

**Published:** 2022-07-22

**Authors:** I Brus, E Speklé, P P Kuijer, M Hardenberg, P Coenen

**Affiliations:** Department of Public and Occupational Health, Amsterdam Public Health Research Institute, Amsterdam UMC, Amsterdam 1081 BT, The Netherlands; Department of Public and Occupational Health, Amsterdam Public Health Research Institute, Amsterdam UMC, Amsterdam 1081 BT, The Netherlands; Arbo Unie, Occupational Health Service, Utrecht 3508 AC, The Netherlands; Department of Public and Occupational Health, Amsterdam Public Health Research Institute, Amsterdam UMC, Amsterdam 1081 BT, The Netherlands; Department of Public and Occupational Health, Amsterdam Public Health Research Institute, Amsterdam UMC, Amsterdam 1081 BT, The Netherlands; Department of Public and Occupational Health, Amsterdam Public Health Research Institute, Amsterdam UMC, Amsterdam 1081 BT, The Netherlands

## Abstract

**Background:**

Low back pain (LBP) is a world leading cause of disability and has substantial impact on individuals and society as a whole. The largest part of the societal burden of LBP is caused by indirect costs, including sick leave.

**Aims:**

We aimed to describe occupational recovery and associated costs for workers consulting an occupational physician (OP) with LBP, and to determine to what extent this differs by diagnoses: non-specific favourable LBP, non-specific unfavourable LBP, lumbosacral radicular syndrome (LRS) and specific LBP.

**Methods:**

We analysed longitudinal dynamic cohort data from an occupational health service, representing ~1.2 million workers from various companies and sectors throughout the Netherlands. The OP registered data on sick leave and LBP diagnoses. A survival analysis was performed on sick leave duration to determine recovery and a linear regression analysis on cost per episode, adjusting for sex, age and working hours.

**Results:**

We analysed 5951 LBP episodes from 5472 workers who consulted an OP, with a median and mean duration sick leave of 95 and 151 days, respectively. The probability of not recovering was 82% at 30 days and 10% at 1 year. The mean cost per episode was €15 350. Specific LBP (€22 999; beta (95% confidence interval [CI]): 16 278 (13 325–19 165)) and LRS (€20 111; beta (95% CI): 13 589 (12 527–14 659)) had the longest and most costly episodes, compared to non-specific favourable LBP (€6745; reference group).

**Conclusions:**

With LRS and non-specific unfavourable LBP accounting for over 83% of LBP-associated sick leave costs, the work-directed care of workers with these two diagnoses deserves increased attention.

## Teaser

Among Dutch workers who consulted an occupational physician with low back pain, 82% did not recover within 30 days and 10% not within 1 year. Mean costs per episode were €15 350, with specific low back pain (€22 999) and lumbosacral radicular syndrome (€20 111) being the diagnoses with the longest and most costly episodes. Our findings underline the major (societal) impact of low back pain for workers, especially those with particular diagnosis to which work-directed care should particularly be targeted.

**Key learning points**

What is already known about this subject:

- Low back pain is the world leading cause of disability, and has substantial impact on individuals and society as a whole.
- The largest part of the societal burden of low back pain results from indirect costs, such as from sick leave.
- Previous studies have estimated the course of sick leave for people with low back pain and their associated costs.

What this study adds:

- Good evidence on the duration and costs of sick leave due to low back pain is scarce. There is a lack of evidence from the Dutch context and on studies in which differences between low back pain diagnoses are considered.
- We described occupational recovery and sick leave costs for workers with low back pain who consulted an occupational physician, and determined to what extent recovery and costs differ depending on low back pain diagnosis.
- Among workers who consulted an occupational physician, 82% did not recover within 30 days and 10% not within 1 year. Mean costs per episode were €15 350, with specific low back pain (€22 999) and lumbosacral radicular syndrome (€20 111) being associated with the longest and most costly episodes, compared to non-specific favourable low back pain (€6745).

What impact this may have on practice or policy:

- Our findings underline the major (societal) impact of low back pain for workers who consulted an occupational physician, especially those with lumbosacral radicular syndrome and non-specific unfavourable low back pain.
- This indicates that work-directed care should particularly be targeted to workers with these diagnoses, who deserve increased attention by occupational physicians.
- Evaluating and assessing the treatment of these workers could ultimately contribute to a cost reduction for these workers accounting for the largest societal burden.

## Introduction

Low back pain (LBP) is a leading cause of disability globally [1]. In 2017, 577 million people suffered from LBP and it accounted for ~65 million years lived with disease [2]. The median point prevalence of LBP is estimated 18%, with a median 1-year prevalence of 38% [3]. LBP also has a large societal impact; it is among the top 3 diseases accounting for the highest amount of healthcare spending in the USA [4]. Indirect costs, including costs due to sick leave (absenteeism) and productivity loss at work (presenteeism), are estimated to account for 80–90% of total LBP societal costs [5,6].

Despite the significant impact of LBP, recovery of workers on sick leave due to LBP is insufficiently understood. While 60–70% of LBP patients will return to work within 1 month after sick leave, occupational rehabilitation follows a long trajectory for others [7,8]. In a meta-analysis it was estimated that 7% of workers sick-listed for LBP did not return to work within 6 months [8]. Although previous research indicated that the specific diagnosis may impact the recovery of LBP [9,10], few studies have taken diagnosis into account when evaluating recovery of workers with LBP. Studies usually only differentiate between non-specific and specific LBP, because of which the knowledge on sick leave associated with specific LBP diagnoses is still largely unknown [9]. A systematic review from 2004 stated that there is limited evidence that specific LBP (e.g. hernia nuclei pulposi or spinal stenosis) is associated with prolonged sick leave duration during the first 3 months of sick leave, but that there is limited evidence on sick leave duration after 3 months [10].

Although LBP-associated sick leave is known to cause substantial indirect costs, estimates of these costs from recent studies vary widely, ranging from ~€3000 to ~€16 000 per episode [11,12]. A reason for this variation could be that it is unclear what underlying LBP diagnoses are associated with sick leave costs, as studies either do not differentiate between LBP diagnoses or only focus on one type of LBP [13,14]. Only one known study distinguished several LBP diagnoses [11].

In short, although LBP has a substantial societal impact, evidence on recovery after sick leave due to LBP is lacking. Insight into the extent to which recovery differs depending on the diagnosis is urgently needed to optimize occupational healthcare for LBP, by developing interventions for specific subgroups who are at high risk of prolonged sick leave with substantial associated costs. Therefore, we aimed to describe occupational recovery and sick leave costs for workers with LBP who consulted an occupational physician (OP). We also aimed to determine to what extent recovery and costs differ depending on LBP diagnosis.

## Methods

We conducted analyses on a dynamic longitudinal cohort study based on a database from one of the largest occupational health services in the Netherlands, providing services for ~1.2 million employees and 12 000 organizations in different sectors throughout the country. The database contained information of workers who visited an OP between 2013 and 2019 and these physicians registered personal and medical data. Workers usually visited an OP if they were on sick leave for longer than a week or if they had a high risk of prolonged sick leave, although this depended on the conditions of the contract between the occupational health service and the employer. The database was not set up for research purposes. Due to the large number of included individuals, it was not possible to obtain *post hoc* informed consent. Because of the magnitude of the database and since data were fully anonymized, the Medical Ethical Committee of the Amsterdam UMC (location VUmc) granted permission for this course of action (reference no. 2020.104).

In line with the definition for the employed labour force of Statistics Netherlands, we included workers aged 15–75 years [15]. We excluded workers working <4 h per week as they are likely to be flex-workers or have a zero-hour contract and possibly worked more hours than registered. We excluded workers working >48 h per week as this is the maximum that employees are allowed to work for a prolonged period of time according to Dutch legislation [16]. The original database contained missing information and entry errors. We were unable to impute missing information and only analysed complete cases. Cases that contained entry errors, e.g. where the end date of an episode preceded the start date, were also removed.

Personal data of workers included sex, age (categories: 15–39, 40–49, 50–59 and 60–75 years) and working hours (categories: 4–19, 20–29, 30–39 and 40–48 h per week). Sick leave data included start and end date of an episode, recovery percentage (a percentage of working hours form the contracted time) and diagnosis. LBP diagnoses were coded by OPs using the Classification system for Occupational and Social insurance physicians (CAS), which is based on the International Classification of Diseases (ICD-10) [17]. These codes were categorized into four diagnosis groups in line with a new policy for LBP management that was introduced by the occupational health service in 2020, which is based on the new guideline LBP and LRS published by the Dutch Association of Occupational Medicine [18]. Table 1 shows an overview of CAS codes and categories that were used. In line with the definition of the Dutch Employee Insurance Agency, we applied a maximum duration of 2 years per sick leave episode [19]. Consequently, we only used data of episodes starting between 2015 and 2017, as episodes starting in 2013–14 could have actually started before 2013 and episodes starting in 2018–19 could have lasted past 2019 given the 2-year time frame. As in this study, among other things, we intended to estimate the duration of sick leave episodes, only those episodes that could reach the maximum duration of 2 years were included.

**Table 1.** Categorization of LBP diagnoses

CAS-code	Diagnosis	Category
L101	Acute non-specific LBP (<6 weeks)	Non-specific favourable LBP
L102	Subacute non-specific LBP (6–12 weeks)	Non-specific unfavourable LBP
L103	Chronic non-specific LBP (>12 weeks)	Non-specific unfavourable LBP
L105	Lumbago with sciatica	Non-specific unfavourable LBP
L601	Spondylosis (osteoarthritis of the spine)	Non-specific unfavourable LBP
L622	Other discopathies	Non-specific unfavourable LBP
L104	Sciatica	LRS
L621	Hernia nuclei pulposi L4–L5 or L5–S1	LRS
L625	Radiculopathy	LRS
L600	Ankylosing spondylitis (Bechterew’s disease)	Specific LBP
L602	Spinal stenosis	Specific LBP
L611	Spondylolysis	Specific LBP
L612	Spondylolisthesis	Specific LBP
L613	Juvenile osteochondrosis of the spine (Calvé/Scheuermann’s disease)	Specific LBP
L629	Other conditions of the spine	Specific LBP

Sick leave duration was calculated by estimating the number of work weeks between the start and the end of each sick leave episode. The costs related to sick leave were calculated using the number of working hours per week, the recovery percentage and productivity costs, using sex- and age-specific average wage rates from Statistics Netherlands [20]. We applied the human capital approach to calculate costs as this captures the employers’ perspective and is the most commonly applied method in cost-of-illness studies on LBP [21].

Dutch legislation stipulates that workers will receive at least 70% of their wage and at least the minimum wage during the first year of sick leave and at least 70% during the second year [19]. However, most collective labour agreements state that employers will pay 100% of wages during the first year and, depending on the sector, 75–100% during the second year. As we were unable to determine the exact percentage, we assumed that workers would be payed 100%, which is likely a closer approximation of the true costs of sick leave than assuming 70%.

**Figure 1.**
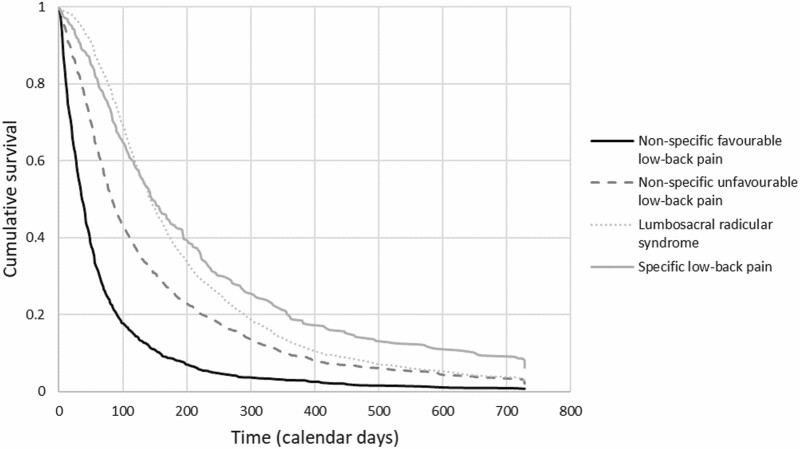
Kaplan-Meier curves showing the cumulative probability of not recovering per diagnosis.

We computed the mean (with 95% confidence interval; 95% CI) and median (with interquartile range; IQR) duration of a sick leave episode in calendar days. We also plotted Kaplan–Meier curves and survival probabilities to determine differences in recovery between diagnoses. A univariable Cox regression analyses with diagnosis as the independent variable and sick leave duration as the dependent variable was conducted. Subsequently, we performed a multivariable regression analysis in which we adjusted for sex, age and working hours, as previous research has shown that these factors could possibly influence the association [22–24]. The assumptions of the model (i.e. proportional hazards, linearity and independence) were checked. Because hazards were non-proportional, we used time-dependent covariates to estimate the hazard ratios (HRs) for two different time periods: 0–150 days and >150 days. This cut-off was chosen as 150 days was the approximate point where Kaplan–Meier curves of different diagnoses cross (Figure 1). We presented HRs with the 95% CI. As in this study, in contrast to many other studies, the outcome was recovery (rather than the incidence of some adverse event), an HR above 1 depicts an increased probability of recovery.

For cost per episode, we performed a univariable linear regression with diagnosis as the independent variable and subsequently a multivariable model to adjust for sex, age and working hours. In these analyses we performed bootstrapping procedures to obtain stable numerical estimates of 95% CI. Bias-corrected and accelerated bootstrapping with 5000 replications was used to deal with positively skewed data distribution [25]. To address heteroscedasticity, we used wild bootstrapping instead of simple bootstrapping [26]. We performed sensitivity analyses in which we also included workers working <4 or >48 h per week, who were excluded in the primary analyses. All analyses were conducted with SPSS version 26. We adopted statistical significance with *P*-values < 0.05.

## Results

Our data set consisted of 14 912 workers with 18 005 sick leave episodes due to back pain who consulted an OP. After data cleaning, 5951 sick leave episodes due to LBP of 5472 workers remained (Supplementary File 1, available as Supplementary data at *Occupational Medicine* Online). Most workers were male (68%), who were responsible for 68% of all sick leave episodes (Table 2). Most workers were 50–59 years old (34%), followed by the 40–49 age category (28%). The majority of participants worked 30–39 h per week (40%), with the second-largest category being 40–48 h per week (35%). LRS was the largest diagnosis group; 38% of episodes fell into this category, followed by non-specific unfavourable LBP (34%).

**Table 2.** Sample characteristics, shown for individual participants as well as per episode

	Per worker	Per episode
Total	*N* = 5472	*N* = 5951
Sex, *n* (%)		
Female	1775 (32%)	1878 (32%)
Male	3697 (68%)	4073 (68%)
Age, *n* (%)		
15–39	1259 (23%)	1351 (23%)
40–49	1534 (28%)	1684 (28%)
50–59	1862 (34%)	2015 (34%)
60–75	817 (15%)	901 (15%)
Working hours per week, *n* (%)		
4–19	640 (12%)	673 (11%)
20–29	727 (13%)	780 (13%)
30–39	2169 (40%)	2343 (39%)
40–48	1936 (35%)	2155 (36%)
Diagnosis group, *n* (%)^a^		
Non-specific favourable LBP		1225 (22%)
Non-specific unfavourable LBP		2055 (35%)
LRS		2281 (38%)
Specific LBP		290 (5%)

^a^Because some workers had multiple episodes in different diagnoses, this could not be calculated per worker.

Mean sick leave duration in calendar days was 151.4 (95% CI 147.3–155.6) and the median was 95.0 (IQR 150.0). The probability of non-recovery was 82% at 30 days, 48% at 100 days, 10% at 1 year and 2% at 2 years (Supplementary File 2, available as Supplementary data at *Occupational Medicine* Online). Workers with non-specific favourable LBP had the fastest recovery, followed by workers with non-specific unfavourable LBP, with non-recovery of 3% and 9%, respectively, at 1 year (Figure 1). Workers with LRS initially recovered slowest. Yet from 150 days onwards, workers with specific LBP had the lowest recovery probability. The mean sick leave duration in calendar days was 68.5 (95% CI 62.9–74.1) for non-specific favourable LBP, 144.8 (137.4–152.2) for non-specific unfavourable LBP, 195.9 (188.8–202.9) for LRS and 225.6 (200.0–252.7) for specific LBP. There were no clear differences in recovery pattern between the six diagnoses in the specific LBP diagnosis category (Supplementary File 3, available as Supplementary data at *Occupational Medicine* Online).

Those with different LBP diagnoses significantly differed in their occupational recovery during the first 150 days (Table 3), with adjusted HR (95% CI) depicting slower recovery of 0.451 (0.417–0.487) for non-specific unfavourable LBP, 0.253 (0.233–0.275) for LRS and 0.258 (0.217–0.306) for specific LBP, compared to non-specific favourable LBP. Beyond 150 days group differences attenuated, with only those with a specific LBP diagnosis having a reduced recovery rate compared to those with non-specific favourable LBP (HR (95% CI): 0.664 (0.520–0.849)). Results for unadjusted and adjusted models were very similar, with <0.01 difference between the models. When including workers working <4 or >48 h per week (*n* = 279), sample characteristics and the effects of the regression models remained relatively the same (Supplementary File 4, available as Supplementary data at *Occupational Medicine* Online).

**Table 3.** Cox regression analysis of the association between diagnoses and the duration of a sick leave episode in calendar days, unadjusted and adjusted for sex, age and working hours

	0–150 days			
	Univariable regression model		Multivariable regression model	
	HR [95% CI]	*P*-value	HR [95% CI]	*P*-value
Diagnosis				
Non-specific favourable LBP	Reference		Reference	
Non-specific unfavourable LBP	0.442 [0.409–0.478]	0	0.451 [0.417–0.487]	0
LRS	0.248 [0.229–0.269]	0	0.253 [0.233–0.275]	0
Specific LBP	0.250 [0.211–0.297]	0	0.258 [0.217–0.306]	0
Sex				
Female	Reference		Reference	
Male	1.643 [1.532–1.763]	0	1.415 [1.303–1.536]	0
Age				
15–39	Reference		Reference	
40–49	0.931 [0.854–1.016]	0.108	0.887 [0.813–0.968]	0.007
50–59	0.941 [0.866–1.023]	0.155	0.917 [0.843–0.998]	0.044
60–75	0.908 [0.819–1.007]	0.067	0.872 [0.785–0.968]	0.01
Working hours per week				
4–19	Reference		Reference	
20–29	0.727 [0.635–0.832]	0	0.839 [0.733–0.961]	0.011
30–39	1.152 [1.036–1.281]	0.009	1.069 [0.956–1.196]	0.243
40–48	1.405 [1.264–1.563]	0	1.239 [1.103–1.393]	0
	>150 days			
	Univariable regression model		Multivariable regression model	
	HR [95% CI]	*P*-value	HR [95% CI]	*P*-value
Diagnosis				
Non-specific favourable LBP	Reference		Reference	
Non-specific unfavourable LBP	0.849 [0.703–1.026]	0.09	0.851 [0.704–1.029]	0.096
LRS	0.957 [0.799–1.148]	0.638	0.960 [0.800–1.151]	0.658
Specific LBP	0.657 [0.514–0.839]	0.001	0.664 [0.520–0.849]	0.001
Sex				
Female	Reference		Reference	
Male	1.054 [0.961–1.156]	0.266	1.023 [0.919–1.139]	0.679
Age				
15–39	Reference		Reference	
40–49	0.954 [0.838–1.085]	0.473	0.971 [0.853–1.106]	0.659
50–59	0.877 [0.776–0.994]	0.04	0.895 [0.788–1.016]	0.087
60–75	0.920 [0.793–1.068]	0.272	0.945 [0.812–1.100]	0.463
Working hours per week				
4–19	Reference		Reference	
20–29	0.959 [0.811–1.135]	0.629	0.949 [0.802–1.123]	0.545
30–39	1.120 [0.964–1.302]	0.137	1.102 [0.941–1.290]	0.229
40–48	1.067 [0.914–1.245]	0.414	1.038 [0.876–1.228]	0.668

Analyses were stratified for the first 150 days of recovery (upper panel) and beyond 150 days of recovery (lower panel).

**Figure 2.**
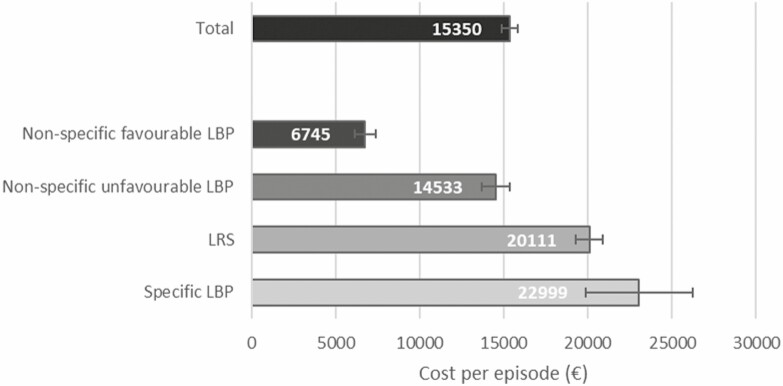
Mean cost per episode (€) with the 95% CI for the total sample and per diagnosis.

The mean cost per LBP episode was €15 350 (Figure 2). Workers with non-specific favourable LBP had the lowest cost per episode (€6745; reference group), followed by workers with non-specific unfavourable LBP (€14 533; adjusted beta (95% CI): 7792 (6748–8819)), followed by LRS (€20 111; beta (95% CI): 13 589 (12 527–14 659)) and specific LBP (€22 999; 16 278 (13 325–19 165)) (Supplementary File 5, available as Supplementary data at *Occupational Medicine* Online).

## Discussion

We aimed to describe occupational recovery and costs of LBP of workers in the Netherlands who consulted an OP, and to determine to what extent this differed by diagnosis. The median duration of an LBP sick leave episode for workers consulting an OP was 95 days and the mean duration was 151 days, with a probability of not recovering of 82% after 30 days and 9% after 1 year. The mean cost of a sick leave episode was €15 350. Workers with non-specific favourable LBP had the shortest and least costly sick leave episodes compared to workers with LRS and specific LBP with the longest and most costly episodes. This indicates that the work-related rehabilitation and care should particularly be targeted to workers with such diagnoses. Recently, a Dutch guideline for LBP and LRS has been developed for occupational and insurance physicians that could aid such care [18]. In addition, recent research shows that the transition rate from acute to chronic LBP is accelerated by guideline non-concordant care and that half of LBP patients receive such care in primary care [27]. This highlights the importance of studying the care received by workers on sick leave due to LRS and non-specific unfavourable LBP and to determine whether the provided treatment is in line with current guidelines.

The duration and costs of LBP-induced sick leave were considerably higher in our study than in previous research. A German study [28] reported €481 and €228 per year in long- and short-term productivity loss due to LBP. In a meta-analysis by Wynne-Jones *et al.*, the median and mean duration ranged from 7–61 and 1–41 days, respectively [8]. A Dutch study reported a median duration of 81 and a mean duration of 122 calendar days for workers with LBP who already were on sick leave [23]. These differences in sick leave duration might be due to several factors. First, workers on sick leave due to LBP who consulted an OP typically have more serious complaints than workers who are recruited in other settings, such as primary healthcare. Workers in our study were typically on sick leave for >1 week before consulting their OP, which may have resulted in an overestimation of the average duration and costs of sick leave of the general Dutch working population. Second, we measured sick leave duration until full return to work, while some aforementioned studies took partial return to work as end point. Third, self-reported sick leave possibly leads to lower estimates of episode duration than registration by OPs. Episodes of 1–7 days might not be registered in our database, as such workers likely return to work before consulting OP. Wynne-Jones *et al.* found that the average duration was shorter in studies using self-reporting compared to registry-based sick leave [8]. Lastly, our data set contained 332 episodes for which recovery was registered to be above 90% at some point during sick leave. These high recovery percentages might not indicate actual sick leave, but ‘administrative’ sick leave. This could have led to an overestimation of sick leave duration. However, as this phenomenon was present for all diagnoses, it is unlikely that it would have influenced between-diagnoses differences.

In a systematic review from 2004 [10], it was concluded that people with specific back disorders had a higher risk of prolonged sick leave than people with non-specific LBP. A Swedish registry-based study found that 11% of non-specific LBP episodes lasted >90 days compared to 65% of disc disorder episodes [9]. These studies are in line with our findings of longest sick leave duration for specific LBP and LRS. Steenstra *et al.* performed a systematic review and found that radiating pain was associated with prolonged sick leave for acute LBP, but that this effect was attenuated in later phases [24]. Our results are in line with this as they indicate that workers with LRS are less likely to return to work than workers with non-specific unfavourable LBP during the first 150 days of sick leave, which attenuated after that.

A strength of our study is that our results are based on a large data set, with high power of the analyses. Secondly, we used data on diagnosis and sick leave episodes registered by OPs instead of self-reported data. While self-reported sick leave seems to correspond reasonably well with registered sick leave, especially for short episodes, it might be a less accurate method to assess long sick leave episodes [29,30]. Finally, our estimations of cost per episode provide insights into the economic impact of LBP-related sick leave on both an individual level and a societal level.

This study also has several limitations. First, we could only estimate costs due to sick leave. Including other cost categories, such as healthcare costs and presenteeism costs, would have led to a more elaborate estimation of the economic impact of LBP among the working population. Nevertheless, as costs due to sick leave make up the majority of total LBP costs (i.e. up to 90%), our analysis still provides an adequate indication of the economic consequences of LBP [6]. Second, we only had access to information on diagnosis, sex, age and working hours and sick leave. Incorporating other variables such as other health factors, socio-economic status and occupational factors (e.g. function, physical work demands and job satisfaction) would have been relevant as previous studies indicated that these factors also affect LBP and return to work [22,24]. There was a substantial proportion of missing data on the available variables (e.g. sickness leave duration and percentage), mainly due to OPs not consistently completing the database information. Although we have no reasons to believe that missing information was systematic (i.e. not at random), it could have biased our findings.

To conclude, our findings show the major (societal) impact due to sick leave among workers with LBP who consulted an OP. This is especially true for workers with LRS and non-specific unfavourable LBP, accounting for over 83% of LBP-associated sick leave costs. This indicates that the work-directed care of workers with these diagnoses deserves increased attention by OPs.

## Supplementary Material

kqac067_suppl_Supplementary_File_1

kqac067_suppl_Supplementary_File_2

kqac067_suppl_Supplementary_File_3

kqac067_suppl_Supplementary_File_4

kqac067_suppl_Supplementary_File_5
