# Supplementary figures and images for: Occupational recovery of Dutch workers with low back pain
Source: Occup Med (Lond). 2022 Jul 22;72(7):462–9. doi: 10.1093/occmed/kqac067 (PMC9578671; doi:10.1093/occmed/kqac067)

**Supplementary file 1. Flowchart depicting the process of data cleaning and data selection**

***
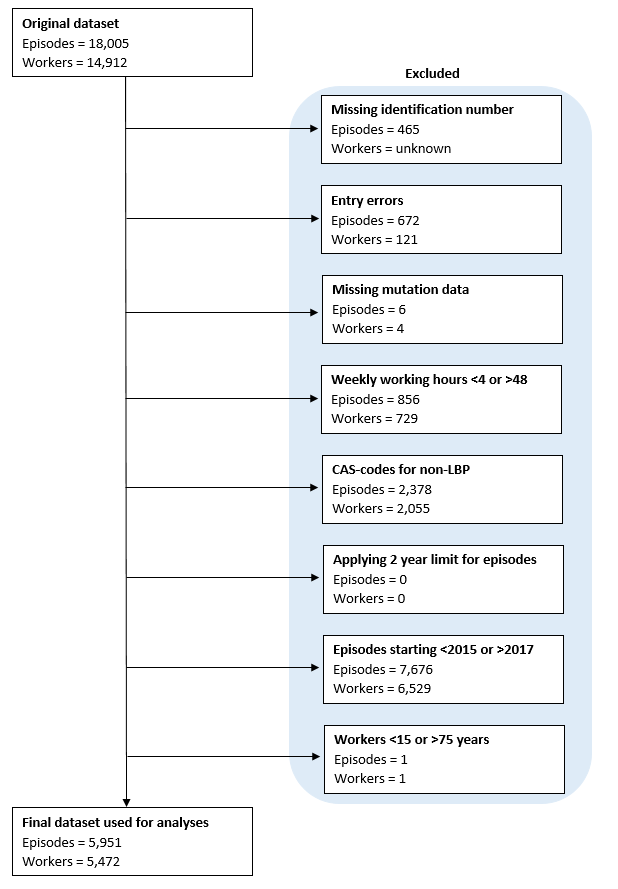
***

Supplement: kqac067_suppl_Supplementary_File_1 [file kqac067_suppl_supplementary_file_1.docx]
