# Supplementary material for: Occupational recovery of Dutch workers with low back pain
Source: Occup Med (Lond). 2022 Jul 22;72(7):462–9. doi: 10.1093/occmed/kqac067 (PMC9578671; doi:10.1093/occmed/kqac067)
Supplement: kqac067_suppl_Supplementary_File_2 [file kqac067_suppl_supplementary_file_2.docx]

**Supplementary file 2. Cumulative probability of not recovering at 30 days, 100 days**, **1 year and 2 years**

***Table 1.*** *Cumulative probability of not recovering at 30 days, 100 days, 1 year and 2 years for the total population.*

|  | Total |
| --- | --- |
| Probability at 30 days | 81.8% |
| Probability at 100 days | 47.8% |
| Probability at 1 year | 9.5% |
| Probability at 2 years | 1.7% |

***Table 2.*** *Cumulative probability of not recovering at 30 days, 100 days, 1 year and 2 years per diagnostic group.*

|  | Non-specific favourable LBP | Non-specific unfavourable LBP | LRS | Specific LBP |
| --- | --- | --- | --- | --- |
| Probability at 30 days | 54.7% | 82.2% | 95.7% | 91.7% |
| Probability at 100 days | 17.6% | 42.2% | 68.4% | 65.5% |
| Probability at 1 year | 2.9% | 9.1% | 12.6% | 18.6% |
| Probability at 2 years | 0.7% | 1.8% | 1.8% | 5.5% |
