# Supplementary material for: Occupational recovery of Dutch workers with low back pain
Source: Occup Med (Lond). 2022 Jul 22;72(7):462–9. doi: 10.1093/occmed/kqac067 (PMC9578671; doi:10.1093/occmed/kqac067)

**Supplementary file 3. Kaplan-Meier curves showing the cumulative probability of not recovering per diagnosis in the specific LBP category.**


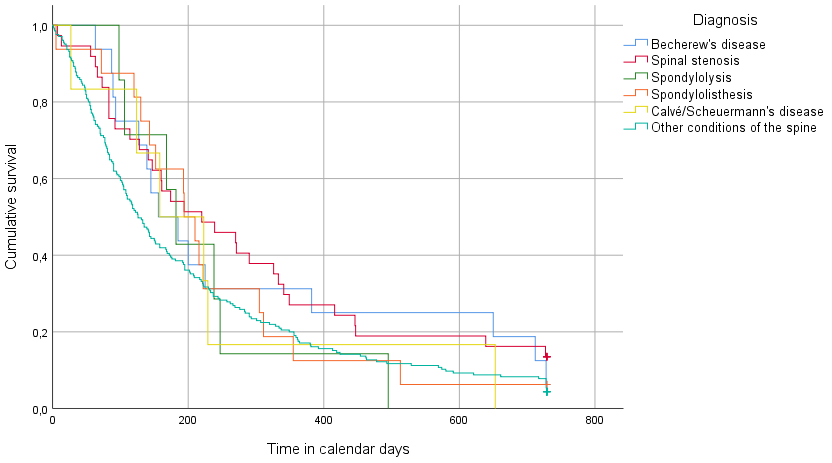

Supplement: kqac067_suppl_Supplementary_File_3 [file kqac067_suppl_supplementary_file_3.docx]
