# Supplementary material for: Occupational recovery of Dutch workers with low back pain
Source: Occup Med (Lond). 2022 Jul 22;72(7):462–9. doi: 10.1093/occmed/kqac067 (PMC9578671; doi:10.1093/occmed/kqac067)
Supplement: kqac067_suppl_Supplementary_File_4 [file kqac067_suppl_supplementary_file_4.docx]

**Supplementary file 4. Sensitivity analysis including workers who worked less than 4 or more than 48 hours per week.**

***Table 1.*** *Population characteristics for workers who work 4-48 hours per week (primary dataset) and for workers of all working hours (including those who work <4 or >48 hours per week)*

|  | Per episode | |
| --- | --- | --- |
|  | **Primary dataset** 4-48 hours | **Extended dataset**  all working hours |
| Total | N = 5951 | N = 6230 |
| Sex, n (%)   - Female - Male | 1878 (31.6%)  4073 (68.4%) | 1964 (31.5%)  4266 (68.5%) |
| Age in categories, n (%)   - 15-39 - 40-49 - 50-59 - 60-75 | 1351 (22.7%)  1684 (28.3%)  2015 (33.9%)  901 (15.1%) | 1418 (22.8%)  1754 (28.2%)  2115 (33.9%)  943 (15.1%) |
| Working hours per week in categories, n (%)   - <4 - 4-19 - 20-29 - 30-39 - 40-48 - >48 | -  673 (11.3%)  780 (13.1%)  2343 (39.4%)  2155 (36.2%)  - | 278 (4.5%)  673 (10.8%)  780 (12.5%)  2343 (37.6%)  2155 (34.6%)  1 (0.0%) |
| Diagnostic group, n (%)*   - Non-specific favourable LBP - Non-specific unfavourable LBP - LRS - Specific LBP | 1225 (22.3%)  2055 (34.5%)  2281 (38.3%)  290 (4.9%) | 1388 (22.3%)  2141 (34.4%)  2398 (38.5%)  303 (4.9%) |

***Table 2.*** *Cox regression analysis of the association between diagnostic group and the duration of a sick leave episode in calendar days, unadjusted and adjusted for sex, age and working hours, for workers of all working hours, including those who work <4 or >48 hours per week (5851 episodes). Analyses were stratified for the first 150 days of recovery (upper panel) and beyond 150 days of recovery (lower panel).*

|  | 0-150 days | | | |
| --- | --- | --- | --- | --- |
|  | **Univariable regression model** | | **Multivariable regression model** | |
|  | HR [95% CI] | p-value | HR [95% CI] | p-value |
| Diagnostic group  Non-specific favourable LBP  Non-specific unfavourable LBP  LRS  Specific LBP | Reference  0.443 [0.410-0.478]  0.246 [0.228-0.267]  0.252 [0.213-0.298] | 0.000  0.000  0.000 | Reference  0.451 [0.418-0.487]  0.251 [0.232-0.272]  0.259 [0.219-0.307] | 0.000  0.000  0.000 |
| Sex  Female  Male | Reference  1.641 [1.532-1.758] | 0.000 | Reference  1.430 [1.322-1.548] | 0.000 |
| Age  15-39  40-49  50-59  60-75 | Reference  0.950 [0.872-1.034]  0.959 [0.883-1.040]  0.926 [0.837-1.025] | 0.225  0.312  0.137 | Reference  0.901 [0.827-0.982]  0.927 [0.854-1.007]  0.887 [0.801-0.983] | 0.018  0.073  0.022 |
| Working hours per week  <20  20-29  30-39  >39 | Reference  0.710 [0.627-0.805]  1.125 [1.025-1.235]  1.371 [1.249-1.506] | 0.000  0.013  0.000 | Reference  0.828 [0.729-0.939]  1.047 [0.951-1.154]  1.212 [1.096-1.340] | 0.003  0.349  0.000 |
|  | **>150 days** | | | |
|  | **Univariable regression model** | | **Multivariable regression model** | |
|  | HR [95% CI] | p-value | HR [95% CI] | p-value |
| Diagnostic group  Non-specific favourable LBP  Non-specific unfavourable LBP  LRS  Specific LBP | Reference  0.834 [0.694-1.003]  0.945 [0.792-1.127]  0.620 [0.487-0.788] | 0.053  0.527  0.000 | Reference  0.838 [0.697-1.009]  0.948 [0.794-1.131]  0.632 [0.497-0.804] | 0.062  0.552  0.000 |
| Sex  Female  Male | Reference  1.043 [0.953-1.141] | 0.363 | Reference  1.008 [0.910-1.117] | 0.872 |
| Age  15-39  40-49  50-59  60-75 | Reference  0.908 [0.801-1.029]  0.846 [0.749-0.955]  0.876 [0.758-1.013] | 0.130  0.007  0.075 | Reference  0.927 [0.816-1.052]  0.871 [0.770-0.985]  0.910 [0.785-1.056] | 0.239  0.027  0.215 |
| Working hours per week  <20  20-29  30-39  >39 | Reference  1.004 [0.863-1.169]  1.173 [1.028-1.338]  1.119 [0.976-1.284] | 0.957  0.018  0.108 | Reference  0.994 [0.853-1.157]  1.160 [1.013-1.330]  1.099 [0.950-1.272] | 0.936  0.032  0.204 |
