# Supplementary material for: Occupational recovery of Dutch workers with low back pain
Source: Occup Med (Lond). 2022 Jul 22;72(7):462–9. doi: 10.1093/occmed/kqac067 (PMC9578671; doi:10.1093/occmed/kqac067)
Supplement: kqac067_suppl_Supplementary_File_5 [file kqac067_suppl_supplementary_file_5.docx]

**Supplementary file 5. Linear regression analysis of the association between diagnostic group and cost per episode, unadjusted and adjusted for sex, age and working hours. Sick leave duration in calendar days and cost per episode are also shown per subgroup.**

|  | Sick leave duration (calendar days) | Cost (€) | Univariable regression model | | Multivariable regression model | |
| --- | --- | --- | --- | --- | --- | --- |
|  | Mean [95% CI] | Mean [95% CI] | Beta [95% CI] | p-value | Beta [95% CI] | p-value |
| Total | 151.4 [147.3-155.6] | 15,350 [14,855-15,844] |  |  |  |  |
| Diagnostic group  Non-specific favourable LBP  Non-specific unfavourable LBP  LRS  Specific LBP | 68.5 [62.9-74.1]  144.8 [137.4-152.2]  195.9 [188.8-202.9]  225.6 [200.0-252.7] | 6,745 [6,112-7,409]  14,533 [13,710-15,348]  20,111 [19,294-20,906]  22,999 [19,875-26,249] | Reference  7,788 [6,664-7,393]  13,366 [12,279-14,480]  16,254 [13,173-19,360] | 0.000  0.000  0.000 | Reference  7,792 [6,748-8,819]  13,589 [12,527-14,659]  16,278 [13,325-19,165] | 0.000  0.000  0.000 |
| Sex  Female  Male | 188.0 [179.9-195.6]  134.5 [129.6-139.5] | 13,236 [12,535-13,955]  16,324 [15,662-16,979] | Reference  3,089 [2,117-4,062] | 0.000 | Reference  853 [-323-2,024] | 0.150 |
| Age  15-39  40-49  50-59  60-75 | 142.2 [134.1-150.3]  151.4 [143.5-159.5]  154.6 [147.0-162.3]  157.6 [146.5-169.3] | 11,396 [10,525-12,232]  16,363 [15,396-17,338]  16,511 [15,615-17,362]  16,788 [15,416-18,218] | Reference  4,967 [3,630-6,285]  5,115 [3,863-6,364]  5,392 [3,837-6,984] | 0.000  0.000  0.000 | Reference  4,005 [2,843-5,188]  4,117 [2,970-5,269]  4,625 [3,165-6,095] | 0.000  0.000  0.000 |
| Working hours per week  4-19  20-29  30-39  40-48 | 168.1 [155.6-181.0]  206.8 [193.3-219.8]  146.9 [140.4-153.5]  130.9 [124.1-137.7] | 6,025 [5,441-6,619]  14,472 [13,415-15,500]  16,455 [15,688-17,272]  17,378 [16,366-18,362] | Reference  8,447 [7,273-9,610]  10,431 [9,420-11,383]  11,353 [10,265-12,505] | 0.000  0.000  0.000 | Reference  7,190 [6,011-8,440]  9,657 [8,566-10,741]  10,883 [9,503-12,203] | 0.000  0.000  0.000 |
